# Unraveling the Impact of Serum Zinc Levels on Chronic Kidney Disease: Machine Learning and SHAP Value Interpretation

**DOI:** 10.1002/fsn3.72170

**Published:** 2026-07-31

**Authors:** Xiaoxin Liu, Kexin Zhao, Ningxu Li, Yanyan Liu

**Affiliations:** ^1^ Division of Nephrology Tongji Hospital, Tongji Medical College, Huazhong University of Science and Technology Wuhan Hubei China; ^2^ Department of Nephrology, Liyuan Hospital, Tongji Medical College Huazhong University of Science and Technology Wuhan Hubei China; ^3^ Department of Hematology, The Second Xiangya Hospital Central South University Changsha Hunan China

**Keywords:** chronic kidney disease, machine learning, NHANES, serum zinc, SHAP

## Abstract

Zinc is an essential trace element involved in antioxidant defense, immune regulation, and metabolic homeostasis, but its association with chronic kidney disease (CKD) in the general population remains unclear. We investigated the association between serum zinc levels and prevalent CKD among U.S. adults and explored the predictive relevance of serum zinc using machine‐learning approaches. We conducted a cross‐sectional analysis of adults from NHANES 2011–2016. Prevalent CKD was defined as estimated glomerular filtration rate < 60 mL/min/1.73 m^2^ and/or urinary albumin‐to‐creatinine ratio ≥ 30 mg/g. Survey‐weighted logistic regression models, restricted cubic spline analysis, and prespecified subgroup analyses were performed. Exploratory machine‐learning analyses were conducted using a temporal split, and SHAP was used for model interpretation. A total of 4192 participants were included, of whom 685 had prevalent CKD. In the fully adjusted model, each 1 μmol/L increase in serum zinc was associated with lower odds of prevalent CKD (OR = 0.93, 95% CI: 0.87–0.99). Compared with the lowest quartile, the fully adjusted ORs were 0.65, 0.69, and 0.56 for the second, third, and highest quartiles, respectively (*p* for trend = 0.012). A nonlinear association was observed (*p* for nonlinear = 0.023), with an apparent turning point at approximately 12.29 μmol/L. In exploratory machine‐learning analyses, random forest showed the highest temporal test‐set AUC, while SHAP analysis suggested that serum zinc contributed meaningful information within the predictive framework. Higher serum zinc levels were associated with a lower prevalence of CKD in U.S. adults. Serum zinc may be a relevant biomarker associated with kidney health, although temporality and causality cannot be inferred.

## Graphical abstract

Using nationally representative NHANES 2011–2016 data, this cross‐sectional study included 4192 U.S. adults, of whom 685 had prevalent chronic kidney disease (CKD). Survey‐weighted logistic regression showed that higher serum zinc levels were associated with lower odds of prevalent CKD. Each 1 μmol/L increase in serum zinc was associated with an odds ratio of 0.93 (95% CI, 0.87–0.99), while participants in the highest quartile had lower odds than those in the lowest quartile (OR, 0.56; 95% CI, 0.37–0.84; *p* for trend = 0.012). Restricted cubic spline analysis indicated a significant nonlinear association. In exploratory machine‐learning analyses, random forest achieved a temporal test‐set AUC of 0.7987. These findings suggest that serum zinc may be a relevant biomarker associated with kidney health, although causality cannot be inferred.

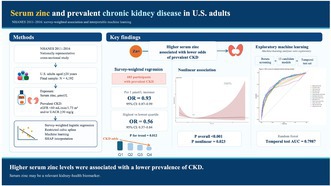

**Abbreviations**
AUC: area under the curve
BMI: body mass index
CI: confidence interval
CKD: chronic kidney disease
CVD: cardiovascular disease
DCA: decision curve analysis
eGFR: estimated glomerular filtration rate
ML: machine learning
NHANES: National Health and Nutrition Examination Survey
OR: odds ratio
PIR: poverty‐income ratio
RCS: restricted cubic spline
ROC: receiver operating characteristic
SD: standard deviation
SHAP: SHapley Additive exPlanations
SZ: serum zinc
UACR: urinary albumin‐to‐creatinine ratio
XGBoost: extreme gradient boosting

## 1 Background

Chronic kidney disease (CKD) is a major global public health challenge that imposes substantial morbidity, mortality, and healthcare costs worldwide (Herrington et al. 2026; KDIGO 2024; Kovesdy 2022). Because established CKD is often progressive and difficult to reverse, the identification of modifiable exposures remains a priority for prevention and early intervention (KDIGO 2024; Kalantar‐Zadeh et al. 2021). In recent years, nutritional factors have attracted increasing attention in CKD research, particularly micronutrients involved in oxidative stress, inflammation, and metabolic regulation (KDIGO 2024; Fukasawa et al. 2023). Zinc is an essential trace element that serves as a structural, catalytic, and regulatory component of more than 300 enzymes and numerous transcription factors, and it plays a central role in antioxidant defense, immune function, and cellular homeostasis (Fukasawa et al. 2023; Chen et al. 2024). Notably, zinc deficiency is common in patients with CKD and has been linked to inflammation, nutritional impairment, anemia, and cardiovascular complications, suggesting that zinc‐related nutritional exposure may be relevant to kidney health (Chen et al. 2024; Nakatani et al. 2021; Lobo et al. 2010; Elgenidy et al. 2023). As a readily measurable biomarker, serum zinc may therefore provide additional insight into the relationship between micronutrient status and kidney health.

Accumulating evidence suggests that zinc is important for metabolic homeostasis, and zinc deficiency has been implicated in a variety of pathological conditions (Chen et al. 2024; Marreiro et al. 2017). In chronic cardiometabolic conditions, abnormal zinc status has been associated with adverse clinical outcomes, underscoring the potential importance of zinc homeostasis in long‐term disease progression (Zhang et al. 2023; Knez and Glibetic 2021). Clinical and nutritional studies have also suggested that zinc status may be relevant to metabolic regulation. For example, zinc supplementation has been associated with improved glycemic control in certain chronic metabolic conditions (Capdor et al. 2013), and adequate dietary zinc intake has been linked to more favorable blood pressure and glycemic profiles in Chinese adults (Wang et al. 2018). Taken together, these findings support the biological relevance of zinc in systemic metabolic regulation and provide a rationale for examining its potential role in kidney health. However, evidence on the association between serum zinc levels and CKD remains limited, particularly in nationally representative U.S. adults, because much of the available literature has been derived from specific clinical settings or non‐U.S. populations.

In this study, we used NHANES 2011–2016 data to examine the association between serum zinc levels and prevalent CKD among U.S. adults using survey‐weighted regression analyses. We further conducted exploratory machine learning analyses to assess whether serum zinc contributed meaningful information within the predictive framework.

## 2 Methods

### 2.1 Study Population

**FIGURE 1:**
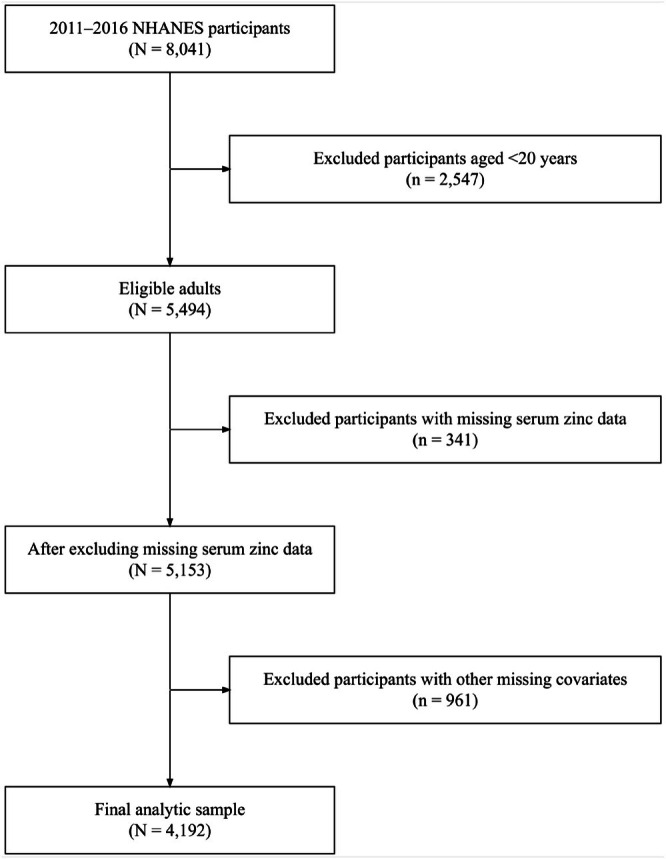
Flowchart of participant selection from NHANES 2011–2016.

Data for this cross‐sectional study were obtained from three consecutive cycles of the National Health and Nutrition Examination Survey (NHANES) (2011–2012, 2013–2014, and 2015–2016). NHANES is a nationally representative survey of the civilian, noninstitutionalized U.S. population conducted by the National Center for Health Statistics (NCHS) using a complex, multistage probability sampling design. Participants complete standardized household interviews and subsequently undergo physical examinations and laboratory assessments in mobile examination centers. All NHANES data used in this study are publicly available from the NCHS website (https://www.cdc.gov/nchs/nhanes/index.htm). The NHANES study protocol was approved by the NCHS Research Ethics Review Board, and all participants signed a written informed consent form prior to data collection (Vital and Health Statistics, Series 1, Programs and Collection Procedures 1994; Zipf et al. 2013). Participants were drawn from three consecutive NHANES cycles (2011–2012, 2013–2014, and 2015–2016). According to the prespecified inclusion and exclusion criteria shown in Figure 1, a total of 4192 U.S. adults were included in the final analytic sample.

### 2.2 Definition of CKD

Prevalent CKD was operationally defined for epidemiologic analysis using a single‐visit estimated glomerular filtration rate (eGFR) < 60 mL/min/1.73 m^2^ and/or a urinary albumin‐to‐creatinine ratio (UACR) ≥ 30 mg/g (KDIGO 2024; Liu et al. 2024; Zhou et al. 2024). Serum creatinine was measured according to the NHANES laboratory protocol, and eGFR was calculated from serum creatinine using the 2021 CKD‐EPI creatinine equation without race (Inker et al. 2021). This equation incorporates serum creatinine, age, and sex, but does not include race. Urinary albumin and urinary creatinine were measured in spot urine samples, and UACR was calculated accordingly. Because NHANES is a cross‐sectional survey and does not provide repeated kidney measurements over at least 3 months, this definition should be interpreted as an operational definition of prevalent CKD rather than a clinically confirmed diagnosis of CKD according to KDIGO chronicity criteria (KDIGO 2024).

### 2.3 Assessment of Serum Zinc Levels

Serum zinc levels were measured in NHANES serum specimens using inductively coupled plasma dynamic reaction cell mass spectrometry (ICP‐DRC‐MS), according to standardized NHANES laboratory procedures (Gau et al. 2021; Huang et al. 2023). Laboratory analyses were performed under established quality control procedures (Gau et al. 2021; Huang et al. 2023).

### 2.4 Definition of Covariates

Covariates were selected a priori according to clinical relevance, nutritional considerations, and data availability in NHANES (Zeng et al. 2026; Gembillo et al. 2026). These variables included demographic characteristics, socioeconomic factors, lifestyle factors, comorbid conditions, dietary exposure, trace‐element status, and medication use. Specifically, the covariates comprised age, sex, race/ethnicity, education level, poverty‐income ratio (PIR), body mass index (BMI), alcohol consumption status, smoking status, diabetes mellitus, cardiovascular disease (CVD), hypertension, hyperlipidaemia, dietary zinc intake, serum copper, ACE inhibitor use, and diuretic use.

Age was analyzed as a continuous variable. Sex was categorized as male or female. Race/ethnicity was classified as Mexican American, Non‐Hispanic Black, Non‐Hispanic White, Other Hispanic, and Others. Education level was categorized as < high school, high school, and > high school. PIR was categorized into three groups: < 1.3, 1.3 to < 3.5, and ≥ 3.5. BMI was calculated as weight in kilograms divided by height in meters squared and categorized as < 25, 25 to < 30, and ≥ 30 kg/m^2^.

Alcohol consumption status was categorized as drinker, nondrinker, and unknown. Smoking status was classified as never, former, and current. Diabetes mellitus, CVD, hypertension, and hyperlipidaemia were each treated as binary variables (yes/no). Dietary zinc intake was derived from the NHANES Day 1 dietary recall and analyzed as a continuous variable. Serum copper was analyzed as a continuous laboratory variable. ACE inhibitor use and diuretic use were defined as binary variables (yes/no) according to medication‐use information collected in NHANES.

### 2.5 Statistical Analysis

#### 2.5.1 Descriptive Analyses, Survey‐Weighted Regression, and Sensitivity Analysis

All statistical analyses were conducted in R software version 4.3.2. The primary epidemiologic analyses were performed using the survey package to account for the complex multistage sampling design of NHANES. For the combined 2011–2016 serum subsample, a merged analytic weight was constructed as WTSA2YR/3, and survey design objects were specified using the NHANES primary sampling units, strata, and analytic weights.

The primary analyses were conducted using a complete‐case approach. Participants with missing values in the primary exposure, outcome‐defining variables, analytic weights, or selected covariates were excluded from the final analytic sample. Continuous variables were summarized as weighted means with standard errors (SEs), and categorical variables were summarized as unweighted counts together with weighted proportions. Differences between participants with and without CKD were compared using survey‐weighted *t*‐tests for continuous variables and Rao–Scott‐adjusted chi‐square tests for categorical variables.

The association between serum zinc and prevalent CKD was evaluated using survey‐weighted logistic regression models. Serum zinc was analyzed both as a continuous variable and as quartiles. Three progressively adjusted models were constructed. Model 1 was unadjusted. Model 2 was adjusted for age, sex, race/ethnicity, education level, and poverty‐income ratio (PIR). Model 3 was further adjusted for BMI, alcohol consumption, smoking status, diabetes mellitus, cardiovascular disease (CVD), hypertension, hyperlipidaemia, dietary zinc intake, serum copper, ACE inhibitor use, and diuretic use. Odds ratios (ORs) and 95% confidence intervals (CIs) were reported.

To assess linear trend across serum zinc quartiles, the median serum zinc value of each quartile was assigned to all participants within that quartile and entered as a continuous term in the fully adjusted survey‐weighted logistic regression model. To evaluate the stability and interpretability of the fully adjusted model, multicollinearity diagnostics based on generalized variance inflation factors (GVIFs) and the events per variable (EPV) were additionally calculated, and these results are presented in the Supporting Information.

Because inflammation may confound the association between serum zinc and prevalent CKD, a survey‐weighted sensitivity analysis restricted to NHANES 2015–2016 was additionally performed, in which hs‐CRP, a high‐sensitivity assay of C‐reactive protein, was entered as an additional covariate.

#### 2.5.2 Restricted Cubic Spline and Subgroup Analyses

To examine the dose–response relationship between serum zinc and prevalent CKD, we fitted survey‐weighted restricted cubic spline (RCS) models with three knots placed at the 10th, 50th, and 90th percentiles of serum zinc (Desquilbet and Mariotti 2010). The overall association and potential nonlinearity of the spline term were additionally evaluated, and the corresponding *p* for overall and *p* for nonlinear values were reported.

Prespecified subgroup analyses were conducted according to age (< 65/≥ 65 years), sex, PIR (< 1.3/1.3 to < 3.5/≥ 3.5), BMI (< 25/25 to < 30/≥ 30 kg/m^2^), smoking status, diabetes mellitus, hyperlipidaemia, and hypertension. Within each subgroup, survey‐weighted logistic regression models were fitted using the same general adjustment framework as the primary analyses, except that the stratification variable itself was not entered as a covariate. Potential heterogeneity was assessed by adding interaction terms between serum zinc and each subgroup variable in the survey‐weighted regression models, and *p* for interaction values were reported.

#### 2.5.3 Exploratory Machine‐Learning Workflow and Validation

The machine‐learning (ML) analyses were treated as exploratory and supplementary, whereas the primary inferential conclusions of the study were based on the survey‐weighted regression analyses. To reduce data leakage and improve the rigor of model evaluation, the ML dataset was partitioned using a temporal split, with participants from NHANES 2011–2012 and 2013–2014 used as the training set and those from NHANES 2015–2016 used as the test set. This test set was used as a temporal validation set, but not as an external dataset.

Because most standard ML algorithms do not directly support the full NHANES complex survey design object, including PSU and strata, these design components were not directly incorporated into the core ML fitting procedures. Instead, the original participant‐level analytic weights were retained after partitioning and carried forward as w_train and w_test. These weights were incorporated where algorithmically feasible and in selected validation summaries, but the ML analyses were not intended to generate separate design‐based nationally representative estimates for the training and test subsets.

Feature selection was performed using the Boruta algorithm, and importantly, Boruta was applied only to the training set. The selected features were then used for downstream model development. Hyperparameter tuning and cross‐validation were likewise conducted within the training set only, whereas the temporal test set was reserved exclusively for final model evaluation. This revised workflow was used to minimize data leakage.

We compared 13 candidate algorithms, including logistic regression, linear discriminant analysis (LDA), quadratic discriminant analysis (QDA), neural network, random forest, ranger, radial support vector machine, bagging, naive Bayes, k‐nearest neighbors (KNN), classification and regression tree (CART), C5.0, and XGBoost. Model discrimination was evaluated using the area under the receiver operating characteristic curve (AUC). Because CKD prevalence was relatively low, model performance was not judged by AUC alone. Additional metrics included accuracy, sensitivity, specificity, precision, F1 score, Cohen's kappa, and weighted Brier score. The best‐performing model was selected according to the temporal test‐set AUC.

For XGBoost, hyperparameter tuning was performed within the training set using grid search combined with 5‐fold cross‐validation. The candidate grid included learning rate, maximum tree depth, minimum child weight, subsample ratio, and column subsample ratio per tree. The final number of boosting rounds was selected according to cross‐validation performance. Because the outcome was imbalanced, class imbalance in XGBoost was addressed using scale_pos_weight, whereas synthetic oversampling methods such as SMOTE were not applied.

Model validation was performed using a three‐part framework consisting of discrimination, calibration, and decision‐curve analysis (DCA) (Vickers and Elkin 2006). Discrimination was assessed by ROC curves and AUC. Calibration was visually assessed using calibration curves and quantitatively summarized using the weighted Brier score. Calibration intercept and slope were additionally explored using weighted logistic recalibration; however, when this procedure produced numerically unstable or non‐informative outputs, these metrics were not retained in the final report. DCA was evaluated over a restricted threshold probability range of 0.01 to 0.40, representing a more plausible low‐to‐moderate risk range for exploratory evaluation in this setting. The best‐performing model was selected according to the temporal test‐set AUC for validation analyses, whereas XGBoost was additionally retained as an exploratory interpretability model for SHAP‐based feature attribution.

#### 2.5.4 Interpretable Tools for Machine Learning

To improve the interpretability of the exploratory ML analysis, we used SHapley Additive exPlanations (SHAP) as a feature‐attribution framework (Lundberg et al. 2020). SHAP is derived from Shapley values in cooperative game theory and provides a consistent method for quantifying the contribution of each feature to model predictions by decomposing the model output into feature‐level contributions.

Because exact computation of Shapley values can be computationally intensive, this study used the efficient implementation available for tree‐based models. In the revised workflow, XGBoost was retained as an exploratory interpretability model, and SHAP values were used to summarize both the global importance and the sample‐level contribution patterns of the included predictors. The SHAP results were interpreted as model‐based feature attribution, rather than as evidence of causal importance or clinical superiority.

All tests were two‐sided, and a *p* value < 0.05 was considered statistically significant.

## 3 Results

### 3.1 Basic Characteristics of the Study Population

A total of 4192 participants were included in the final analysis, including 685 individuals with prevalent CKD and 3507 without CKD. As shown in Table 1, participants with CKD were older and had lower serum zinc and dietary zinc intake, but higher serum copper and BMI than those without CKD. They were also more likely to be female, to have lower educational attainment and a less favorable poverty‐income ratio distribution, and to have diabetes mellitus, cardiovascular disease, hypertension, hyperlipidaemia, ACE inhibitor use, and diuretic use (all *p* < 0.05). Weighted baseline characteristics are presented in Table 1.

**TABLE 1:** Baseline characteristics of the study population according to prevalent CKD status.

Variable	Level	Overall	No CKD	CKD	*p*
Age, years		47.21 (0.46)	45.32 (0.46)	60.09 (0.95)	< 0.001
Serum zinc, μmol/L		12.54 (0.08)	12.59 (0.08)	12.26 (0.13)	0.024
Dietary zinc intake, mg/day		11.25 (0.11)	11.49 (0.12)	9.64 (0.23)	< 0.001
Serum copper, μmol/L		18.58 (0.14)	18.44 (0.15)	19.58 (0.24)	< 0.001
BMI, kg/m^2^		29.17 (0.17)	29.01 (0.17)	30.31 (0.37)	< 0.001
Sex	Female	2101; 51.5%	1755; 50.5%	346; 58.2%	0.008
	Male	2091; 48.5%	1752; 49.5%	339; 41.8%	
Race/ethnicity	Mexican American	547; 8.0%	466; 8.2%	81; 6.9%	0.003
	Non‐Hispanic Black	881; 10.4%	701; 9.9%	180; 13.7%	
	Non‐Hispanic White	1706; 68.1%	1409; 67.9%	297; 69.2%	
	Other Hispanic	452; 6.0%	391; 6.2%	61; 4.9%	
	Others	606; 7.5%	540; 7.8%	66; 5.4%	
Education level	< High school	846; 13.6%	634; 12.3%	212; 22.7%	< 0.001
	> High school	2426; 65.5%	2125; 67.6%	301; 51.3%	
	High school	920; 20.9%	748; 20.2%	172; 25.9%	
Poverty‐income ratio	< 1.3	1354; 21.7%	1092; 20.7%	262; 28.3%	< 0.001
	≥ 3.5	1302; 42.4%	1168; 44.8%	134; 26.3%	
	1.3 to < 3.5	1536; 35.9%	1247; 34.5%	289; 45.5%	
BMI category	< 25	1225; 29.4%	1049; 29.6%	176; 28.1%	0.002
	≥ 30	1620; 37.7%	1316; 36.6%	304; 45.2%	
	25 to < 30	1347; 32.9%	1142; 33.8%	205; 26.7%	
Alcohol status	Drinker	2907; 75.7%	2449; 76.3%	458; 71.7%	< 0.001
	Nondrinker	1057; 19.5%	850; 18.5%	207; 26.3%	
	Unknown	228; 4.7%	208; 5.2%	20; 2.0%	
Smoking status	Current	813; 18.6%	680; 18.7%	133; 18.3%	< 0.001
	Former	986; 24.6%	771; 23.1%	215; 34.9%	
	Never	2393; 56.7%	2056; 58.2%	337; 46.8%	
Diabetes	No	3438; 85.8%	3013; 88.3%	425; 68.6%	< 0.001
	Yes	754; 14.2%	494; 11.7%	260; 31.4%	
Cardiovascular disease	No	3844; 93.2%	3323; 95.3%	521; 79.0%	< 0.001
	Yes	348; 6.8%	184; 4.7%	164; 21.0%	
Hypertension	No	1971; 50.3%	1826; 53.9%	145; 26.0%	< 0.001
	Yes	2221; 49.7%	1681; 46.1%	540; 74.0%	
Hyperlipidaemia	No	1423; 33.7%	1214; 34.3%	209; 29.0%	0.033
	Yes	2769; 66.3%	2293; 65.7%	476; 71.0%	
ACE inhibitor use	No	3605; 87.6%	3106; 89.5%	499; 74.6%	< 0.001
	Yes	587; 12.4%	401; 10.5%	186; 25.4%	
Diuretic use	No	3633; 88.3%	3152; 90.5%	481; 73.6%	< 0.001
	Yes	559; 11.7%	355; 9.5%	204; 26.4%	

*Note:* Continuous variables are expressed as weighted mean (SE), and categorical variables are expressed as unweighted *n* and weighted %. All analyses accounted for the complex sampling design of NHANES. *p* values were obtained from survey‐weighted *t*‐tests for continuous variables and Rao–Scott‐adjusted chi‐square tests for categorical variables.

### 3.2 Association Between Serum Zinc and Prevalent CKD

As shown in Table 2, higher serum zinc levels were significantly associated with lower odds of prevalent CKD in the unadjusted model and in both adjusted models. In the fully adjusted model, each 1 μmol/L increase in serum zinc was associated with 7% lower odds of prevalent CKD (OR = 0.93, 95% CI: 0.87–0.99, *p* = 0.025). When serum zinc was analyzed by quartiles, compared with participants in the lowest quartile, those in Q2, Q3, and Q4 had significantly lower odds of prevalent CKD in the fully adjusted model, with ORs of 0.65 (95% CI: 0.48–0.88), 0.69 (95% CI: 0.50–0.95), and 0.56 (95% CI: 0.37–0.84), respectively. A significant dose–response trend was also observed across quartiles (*p* for trend = 0.012).

**TABLE 2:** Weighted logistic regression analyses of serum zinc levels and odds of prevalent CKD.

Serum zinc levels (μmol/L)	Model 1		Model 2		Model 3	
	OR (95% CI)	*p*	OR (95% CI)	*p*	OR (95% CI)	*p*
Continuous	0.94 (0.89–0.99)	0.023	0.94 (0.88–0.99)	0.039	0.93 (0.87–0.99)	0.025
Q1 [6.26, 10.91]	1 [Reference]		1 [Reference]		1 [Reference]	
Q2 (10.91, 12.29]	0.77 (0.57–1.04)	0.087	0.74 (0.55–0.98)	0.037	0.65 (0.48–0.88)	0.007
Q3 (12.29, 13.82]	0.77 (0.58–1.02)	0.069	0.72 (0.53–0.97)	0.033	0.69 (0.50–0.95)	0.027
Q4 (13.82, 35.57]	0.63 (0.45–0.89)	0.009	0.62 (0.44–0.89)	0.010	0.56 (0.37–0.84)	0.008
*p* for trend		0.011		0.015		0.012

*Note:* Serum zinc was analyzed both as a continuous variable and as quartiles. Model 1: No adjustment. Model 2: adjusted for age, sex, race/ethnicity, education level, and poverty‐income ratio. Model 3: adjusted for education level, PIR, BMI, alcohol consumption, smoking status, diabetes mellitus, CVD, hypertension, hyperlipidemia, dietary zinc, serum copper, ACE inhibitor use and Diuretic use based on Model 2.

To clarify the trend analysis, quartile‐specific median serum zinc values were assigned to participants within each quartile, yielding median values of 9.955, 11.610, 13.020, and 15.040 μmol/L from Q1 to Q4, respectively (Table S1). Using this median‐value modeling approach, the trend variable remained significantly associated with lower odds of prevalent CKD (OR = 0.902, 95% CI: 0.834–0.976, *p* = 0.013; Table S2). Multicollinearity diagnostics showed no evidence of problematic collinearity in the fully adjusted model (Table S3). In addition, the events per variable (EPV) was 27.4, supporting the stability of the regression estimates (Table S4). In the hs‐CRP‐adjusted sensitivity analysis restricted to NHANES 2015–2016 participants with available hs‐CRP data, the inverse association between serum zinc and prevalent CKD remained directionally consistent after additional adjustment for hs‐CRP (continuous serum zinc: OR = 0.990, 95% CI: 0.981–0.997, *p* = 0.037; Table S5).

**FIGURE 2:**
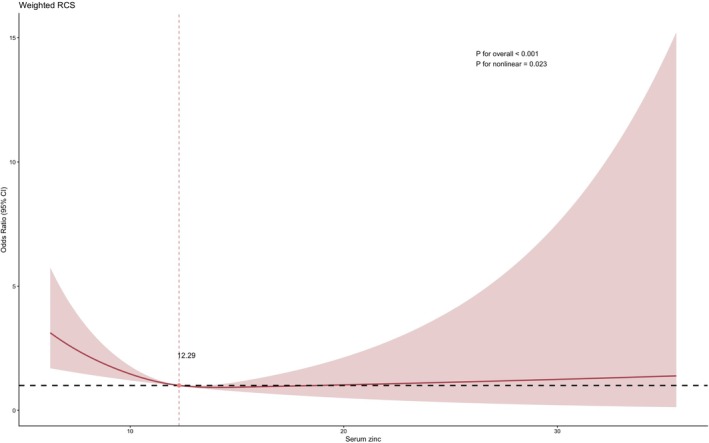
Weighted restricted cubic spline analysis of the association between serum zinc and odds of prevalent CKD. The solid line represents the estimated odds ratio, and the shaded area represents the 95% confidence interval.

The weighted restricted cubic spline analysis further suggested a nonlinear association between serum zinc and the odds of prevalent CKD (Figure 2). The curve showed an apparent turning point at approximately 12.29 μmol/L. Below this level, increasing serum zinc was associated with a marked decrease in the odds of prevalent CKD, whereas above this level the curve became relatively flat, with wider confidence intervals at higher serum zinc concentrations. The overall association was statistically significant (*p* for overall < 0.001), and the test for nonlinearity was also significant (*p* for nonlinear = 0.023).

### 3.3 Subgroup Analysis

**FIGURE 3:**
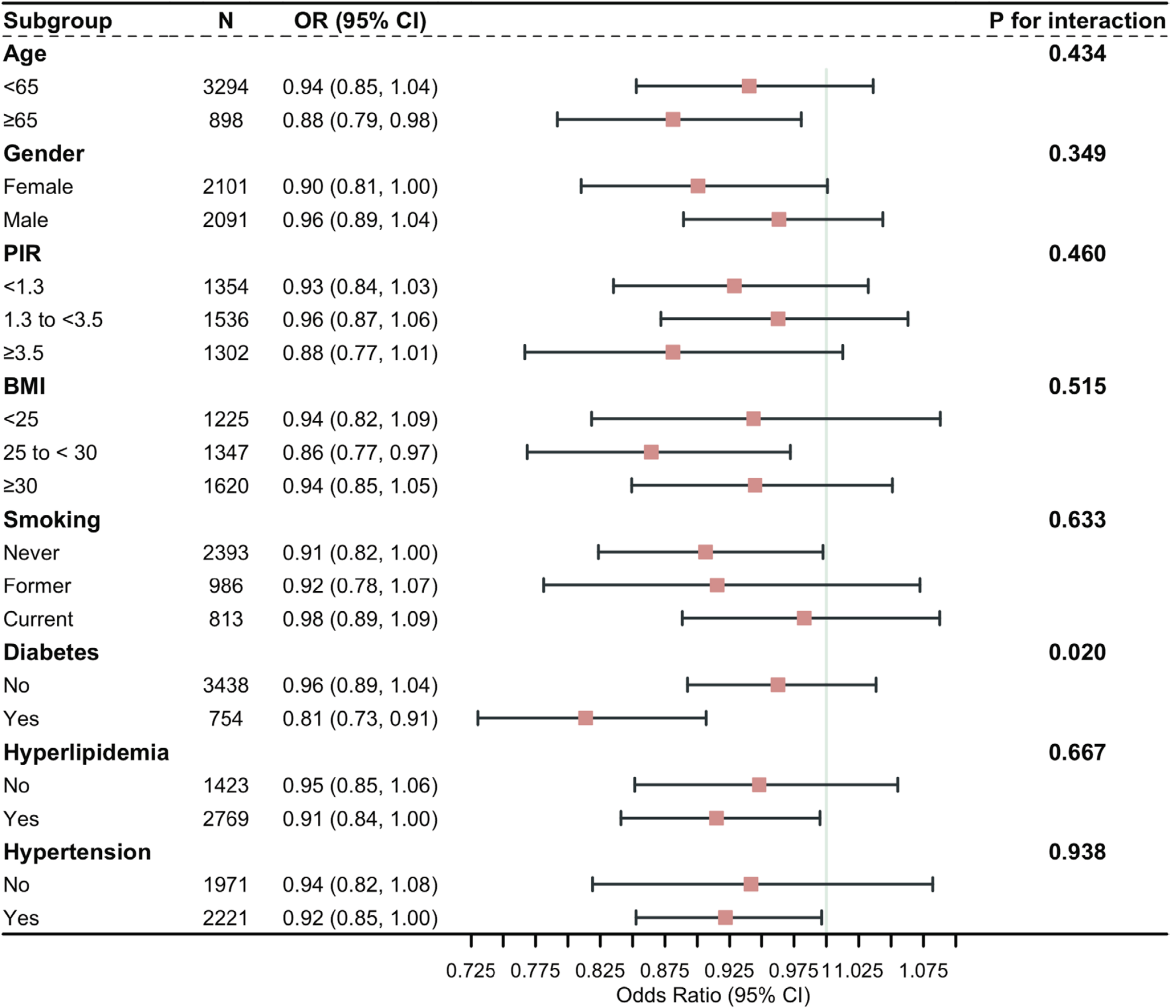
Subgroup analyses of the association between serum zinc and odds of prevalent CKD. Odds ratios and 95% confidence intervals are shown for prespecified subgroups, and *p* values for interaction are presented on the right.

Subgroup analyses were performed according to age, sex, PIR, BMI, smoking status, diabetes mellitus, hyperlipidaemia, and hypertension (Figure 3). Overall, the inverse association between serum zinc and prevalent CKD was generally consistent across most subgroups, and no significant interactions were observed for age, sex, PIR, BMI, smoking status, hyperlipidaemia, or hypertension (all *p* for interaction > 0.05). A significant interaction was observed for diabetes status (*p* for interaction = 0.020). The inverse association between serum zinc and prevalent CKD was stronger among participants with diabetes (OR = 0.81, 95% CI: 0.73–0.91) than among those without diabetes (OR = 0.96, 95% CI: 0.89–1.04).

### 3.4 Boruta‐Based Feature Screening

**FIGURE 4:**
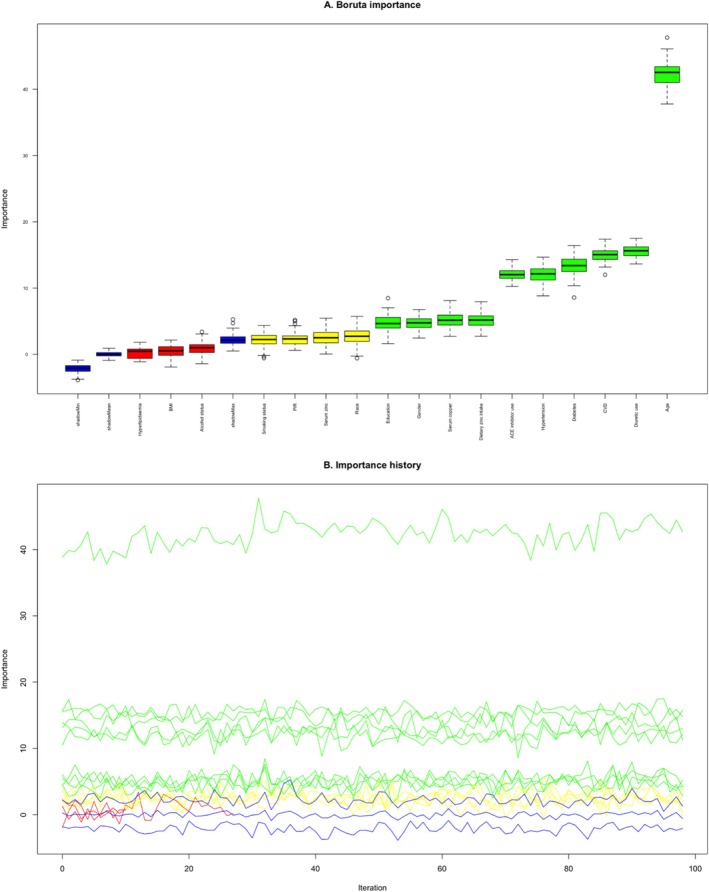
Boruta‐based feature screening for classification of prevalent CKD in the training set. (A) Boruta importance of candidate predictors; (B) Importance history across Boruta iterations.

Boruta‐based feature screening was performed in the training set to support downstream exploratory machine‐learning modeling (Figure 4). The retained features mainly covered demographic, nutritional, cardiometabolic, and medication‐related domains. Among them, age showed the highest Boruta importance, while dietary zinc intake and serum zinc were also retained among the more important predictors. These selected features were subsequently used for model development under the revised leakage‐controlled workflow.

### 3.5 Comparison of Machine‐Learning Model Performance

**FIGURE 5:**
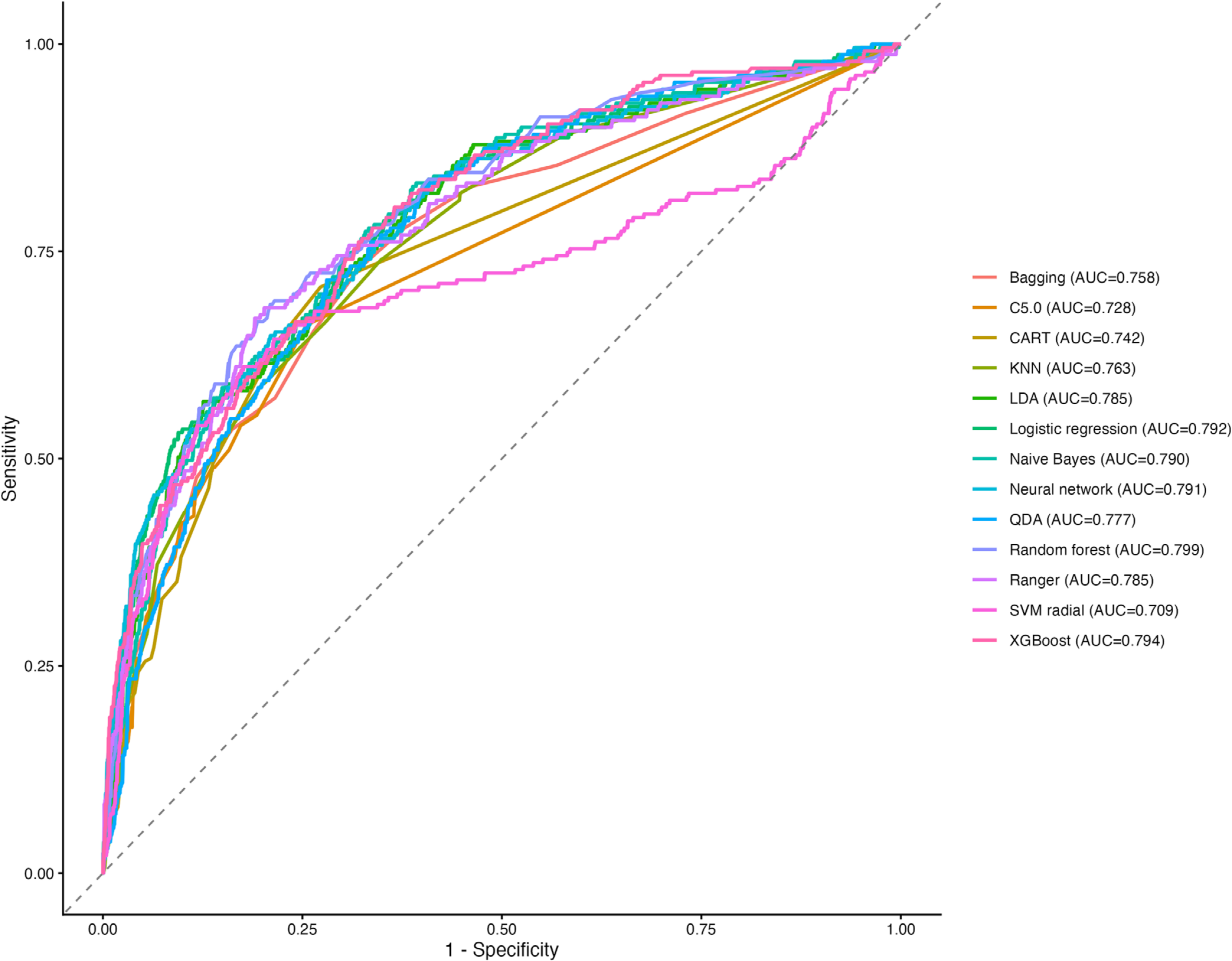
Comparison of machine‐learning model performance for classification of prevalent CKD in the temporal test set. Receiver operating characteristic (ROC) curves and corresponding area under the curve (AUC) values are shown for the compared models.

A total of 13 candidate machine‐learning models were compared in the internal/training set and temporal test set (Figure 5; Table S6). In the temporal test set, the random forest model achieved the highest discrimination (AUC = 0.7987), followed by XGBoost (AUC = 0.7935), logistic regression (AUC = 0.7923), and neural network (AUC = 0.7906). Several other models showed broadly comparable but slightly lower performance. Complete performance metrics are presented in Table S6. In addition, because XGBoost was retained as the exploratory interpretability model for SHAP analysis and was also specifically queried during peer review, its candidate tuning grid, cross‐validation results, and final hyperparameters are summarized in Table S7.

### 3.6 Validation of the Best‐Performing Model

**FIGURE 6:**
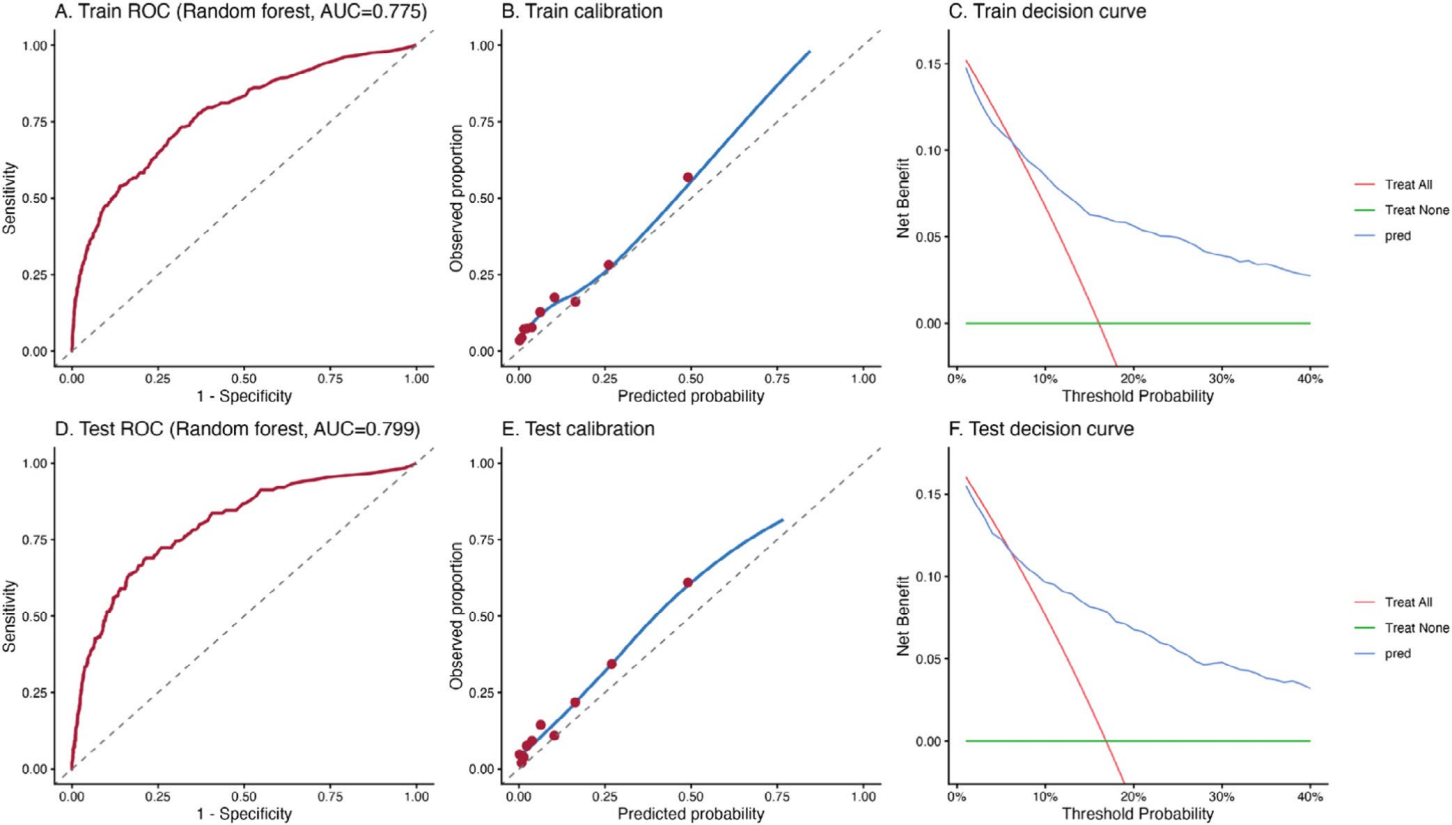
Validation of the best‐performing model (random forest) for classification of prevalent CKD. (A) Training‐set ROC curve; (B) Training‐set calibration curve; (C) Training‐set decision curve analysis; (D) Temporal test‐set ROC curve; (E) Temporal test‐set calibration curve; (F) Temporal test‐set decision curve analysis. Decision curve analysis was evaluated over threshold probabilities from 0.01 to 0.40.

Because the random forest model showed the highest temporal test‐set AUC, it was selected as the best‐performing model for further validation (Figure 6). As shown in Table S8A, the random forest model achieved an internal/training‐set AUC of 0.7753 and a temporal test‐set AUC of 0.7987. In the temporal test set, the model showed a sensitivity of 0.1715, specificity of 0.9830, precision of 0.6721, F1 score of 0.2733, kappa of 0.2198, and a weighted Brier score of 0.0966. The corresponding validation curves are presented in Figure 6.

### 3.7 SHAP Interpretation of the XGBoost Model

**FIGURE 7:**
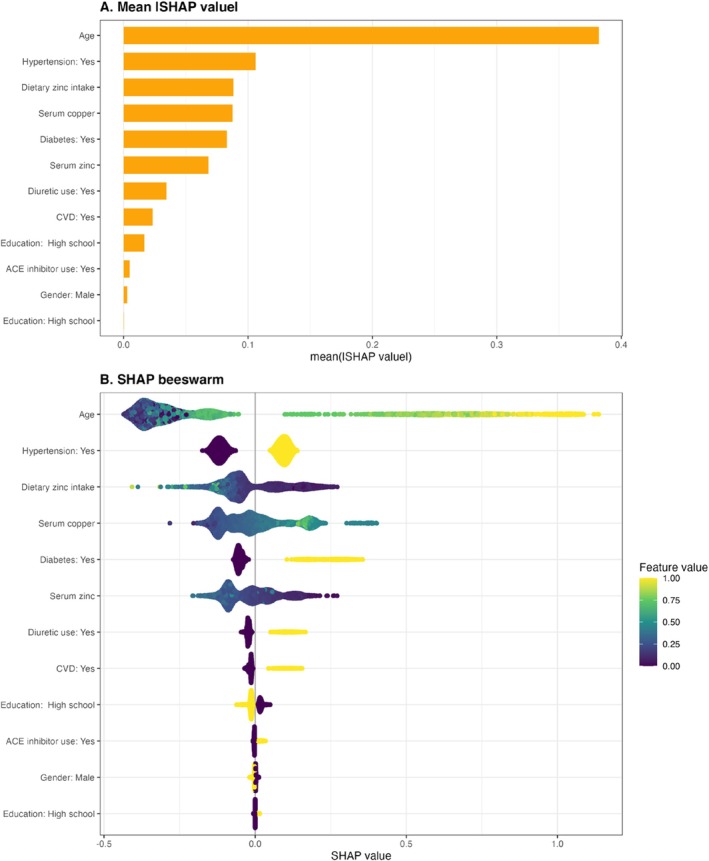
SHAP interpretation of the XGBoost model in the temporal test set. (A) Mean absolute SHAP values showing global feature importance; (B) SHAP beeswarm plot showing the distribution of feature contributions across individuals.

Although XGBoost was not the best‐performing model by temporal test‐set AUC, it was additionally retained for SHAP analysis because it showed comparable discrimination and allowed more straightforward model‐based feature attribution (Figure 7). As shown in Table S8B, XGBoost achieved a test‐set AUC of 0.7935 and a weighted Brier score of 0.0955. The SHAP results indicated that age was the most influential feature within the XGBoost model, followed by dietary zinc intake and serum zinc. Other relatively important contributors included hypertension, serum copper, and diabetes. Overall, these results suggest that serum zinc contributed meaningful information within the exploratory predictive framework.

## 4 Discussion

In this cross‐sectional study based on nationally representative NHANES 2011–2016 data, we found that higher serum zinc levels were independently associated with a lower prevalence of CKD among U.S. adults after adjustment for multiple demographic, lifestyle, and clinical covariates. This association remained generally stable when serum zinc was analyzed in different forms, supporting the robustness of the observed relationship. In the sensitivity analysis restricted to NHANES 2015–2016, additional adjustment for hs‐CRP attenuated the magnitude of the association but did not materially change its inverse direction, suggesting that the serum zinc‐CKD association was not fully explained by systemic inflammation as captured by hs‐CRP. As a clinically measurable indicator of zinc status, serum zinc may provide additional insight into the relationship between micronutrient status and kidney health at the population level. In addition to the conventional regression analyses, our machine‐learning analyses further suggested that serum zinc contributed meaningful information to CKD prediction. Taken together, these findings suggest that serum zinc may be a relevant biomarker associated with CKD prevalence and may provide complementary information for population‐level risk assessment. From a public health perspective, identifying biomarkers associated with CKD may be useful for improving early risk assessment and for better understanding nutritional and metabolic pathways potentially related to kidney health.

Our findings should be interpreted in the context of previous studies on zinc status and kidney‐related outcomes. In a Japanese study of non‐dialysis patients, serum zinc levels tended to decrease with advancing CKD stage and were positively associated with eGFR in univariable analysis, although this association was not retained after multivariable adjustment (Maruyama et al. 2021). In contrast, a study of Chinese adults aged 90 years or older reported that higher plasma zinc levels were associated with lower odds of CKD (Shen et al. 2020), whereas another study from rural Hunan did not confirm an independent association between plasma zinc and reduced renal function after full adjustment (Yang et al. 2019). These differences are not unexpected and may reflect heterogeneity in study populations, zinc biomarkers, kidney outcome definitions, and covariate adjustment strategies. Compared with these earlier reports, our study provides complementary population‐based evidence from a nationally representative sample of U.S. adults and suggests that higher serum zinc levels are associated with lower CKD prevalence in a broader general population setting. This broader population context may be important, because associations observed in clinical or region‐specific samples are not always directly generalizable to the general adult population.

Several biological pathways may help explain the observed inverse association between serum zinc levels and CKD prevalence. Oxidative stress and chronic low‐grade inflammation are central to the development and progression of CKD, and zinc is known to participate in antioxidant defense and immune regulation (Ebert et al. 2021; Li et al. 2014; Bonaventura et al. 2015; Podkowińska and Formanowicz 2020). Experimental studies have shown that zinc deficiency can impair Nrf2‐related antioxidant signaling in renal tubular cells and diabetic kidneys, thereby aggravating oxidative injury, inflammation, and fibrotic changes (Li et al. 2014). In addition, zinc homeostasis has been linked to endothelial integrity as well as glucose and lipid metabolism, all of which are closely involved in microvascular injury and renal function decline (Olechnowicz et al. 2018; McClain et al. 1995). These pathways are relevant to both glomerular and tubular injury in CKD, rather than to kidney‐specific oxidative damage alone. Because diabetes, hypertension, endothelial dysfunction, and metabolic disturbance are major contributors to CKD, the association between serum zinc and kidney health may partly reflect broader vascular and metabolic mechanisms rather than isolated renal processes. These mechanisms provide biological plausibility for the observed association. However, because the present study was cross‐sectional, the findings do not establish temporality, and reverse causation remains possible; lower serum zinc may also reflect nutritional, metabolic, or inflammatory consequences of CKD rather than an etiologic driver of disease.

Beyond the conventional regression analyses, the machine‐learning results provide an additional perspective on the potential relevance of serum zinc in CKD. Rather than replacing traditional epidemiologic models, these approaches may be useful for capturing complex relationships among nutritional, metabolic, and clinical variables in a multifactorial condition such as CKD (Pan and Tong 2024). In this context, the SHAP‐based interpretation improved model transparency by quantifying the relative contribution of serum zinc within the overall prediction framework (Lundberg et al. 2020). However, feature importance in a predictive model should not be interpreted as evidence of causality, and the role of serum zinc in the machine‐learning analyses is better understood as complementary to, rather than stronger than, the association observed in the regression models. Taken together, these findings suggest that serum zinc may have epidemiologic relevance and may contribute meaningful information within exploratory predictive models, although external validation and prospective evaluation are still needed before broader application can be considered (Pan and Tong 2024). This may be particularly relevant in CKD research, where biomarkers are often interrelated with comorbidities and lifestyle factors, making simple linear assumptions insufficient to fully characterize risk patterns.

Several limitations should be acknowledged. First, because this was a cross‐sectional study, the temporal relationship between serum zinc levels and CKD could not be established, and causal inference should therefore be avoided (Wang and Cheng 2020). Second, prevalent CKD was operationally defined using a single reduced eGFR and/or albuminuria measurement in NHANES, rather than a clinically confirmed diagnosis based on persistent abnormalities for at least 3 months (KDIGO 2024). Therefore, the study could not assess CKD chronicity according to KDIGO criteria, and some participants with transient kidney dysfunction, including possible acute kidney injury, or transient albuminuria may have been misclassified. This limitation should be considered when interpreting the findings. Third, serum zinc was measured at a single time point and may not fully reflect long‐term zinc status. Finally, although a wide range of covariates was adjusted for, residual confounding from unmeasured or imprecisely measured factors cannot be excluded. In addition, although the regression analyses fully accounted for the NHANES complex survey design, the ML analyses did not directly incorporate PSU and strata within all model‐fitting procedures and should therefore be interpreted as exploratory and supplementary. Nevertheless, several strengths should also be noted. The use of nationally representative NHANES data enhances the generalizability of the findings to the U.S. adult population, and the standardized laboratory assessment improves measurement consistency. In addition, the combined application of survey‐weighted regression and interpretable machine‐learning methods allowed us to evaluate the association from both inferential and predictive perspectives.

## 5 Conclusion

In conclusion, higher serum zinc levels were associated with a lower prevalence of CKD among U.S. adults in this nationally representative cross‐sectional study. Serum zinc also contributed meaningful information within the exploratory machine‐learning framework. These findings suggest that serum zinc may be a relevant biomarker associated with kidney health at the population level, although prospective studies are needed to clarify temporality and to further evaluate the robustness and broader applicability of this association.

## Author Contributions

**Yanyan Liu:** conceptualization, investigation, visualization, writing – review and editing, methodology, validation, formal analysis, software, resources, supervision. **Xiaoxin Liu:** conceptualization, investigation, writing – original draft, writing – review and editing, visualization, validation, methodology, software, formal analysis. **Kexin Zhao:** conceptualization, investigation, validation, writing – original draft, writing – review and editing, software, data curation, supervision, resources. **Ningxu Li:** conceptualization, methodology, validation, visualization, writing – review and editing.

## Funding

Hubei Provincial Natural Science Foundation of China (2025AFB747).

## Ethics Statement

The NHANES study protocol received approval from the Research Ethics Review Board of the NCHS (Protocol #2011‐17, Protocol #2018‐01).

## Consent

The authors have nothing to report.

## Conflicts of Interest

The authors declare no conflicts of interest.

## Supporting information


**Table S1:** Median serum zinc values assigned to quartiles for the *p* for trend analysis.
**Table S2:** *p* for trend for serum zinc quartiles.
**Table S3:** Multicollinearity diagnostics for the fully adjusted logistic regression model.
**Table S4:** Events per variable for the fully adjusted logistic regression model.
**Table S5:** hs‐CRP‐adjusted sensitivity analysis of serum zinc levels and odds of prevalent CKD restricted to NHANES 2015–2016.
**Table S6:** Performance metrics of the 13 compared machine‐learning models in the internal training and temporal test sets.
**Table S7:** XGBoost tuning procedure and final hyperparameters.
**Table S7A:** Candidate hyperparameter grid for XGBoost.
**Table S7B:** Cross‐validation performance of candidate XGBoost parameter combinations.
**Table S7C:** Final XGBoost hyperparameters used for model fitting.
**Table S8:** Focused validation summaries for the best‐performing model and the XGBoost model used for SHAP analysis.
**Table S8A:** Internal validation metrics of the best‐performing model selected according to the test‐set AUC.
**Table S8B:** Discrimination and calibration metrics of XGBoost used for SHAP analysis.

## Data Availability

The datasets used in this study are all publicly available from NHANES (https://www.cdc.gov/nchs/nhanes/index.htm).
