# Supplementary material for: Unraveling the Impact of Serum Zinc Levels on Chronic Kidney Disease: Machine Learning and SHAP Value Interpretation
Source: Food Sci Nutr. 2026 Jul 31;14(8):e72170. doi: 10.1002/fsn3.72170 (PMC13428217; doi:10.1002/fsn3.72170)
Supplement: Supplementary file 1 — Table S1: Median serum zinc values assigned to quartiles for the p for trend analysis. Table S2: p for trend for serum zinc quartiles. Table S3: Multicollinearity diagnostics for the fully adjusted logistic regression model. Table S4: Events per variable for the fully adjusted logistic regression model. Table S5: hs‐CRP‐adjusted sensitivity analysis of serum zinc levels and odds of prevalent CKD restricted to NHANES 2015–2016. Table S6: Performance metrics of the 13 compared machine‐learning models in the internal training and temporal test sets. Table S7: XGBoost tuning procedure and final hyperparameters. Table S7A: Candidate hyperparameter grid for XGBoost. Table S7B: Cross‐validation performance of candidate XGBoost parameter combinations. Table S7C: Final XGBoost hyperparameters used for model fitting. Table S8: Focused validation summaries for the best‐performing model and the XGBoost model used for SHAP analysis. Table S8A: Internal validation metrics of the best‐performing model selected according to the test‐set AUC. Table S8B: Discrimination and calibration metrics of XGBoost used for SHAP analysis. [file FSN3-14-e72170-s001.docx]

Table S1. Median serum zinc values assigned to quartiles for the P for trend analysis

| **Category** | **Range** | **Median value** |
| --- | --- | --- |
| SZ_Q1 | 6.26–10.91 | 9.955 |
| SZ_Q2 | 10.91–12.29 | 11.610 |
| SZ_Q3 | 12.29–13.82 | 13.020 |
| SZ_Q4 | 13.82–35.57 | 15.040 |

P for trend was calculated by assigning the median serum zinc value of each quartile to all participants in that quartile and entering this variable as a continuous term in the fully adjusted survey-weighted model. This presentation was used to clarify the trend-test methodology and the quartile boundaries.

Table S2. P for trend for serum zinc quartiles

| Variable | OR | 95% CI | P value |
| --- | --- | --- | --- |
| SZ trend | 0.902 | 0.834–0.976 | 0.013 |

The trend test was performed by entering the quartile-specific median serum zinc value as a continuous variable into the fully adjusted survey-weighted logistic regression model.

Table S3. Multicollinearity diagnostics for the fully adjusted logistic regression model

| Variable | Df | GVIF adjusted |
| --- | --- | --- |
| Serum Zinc | 1 | 1.0113 |
| Age | 1 | 1.2274 |
| Gender | 1 | 1.2053 |
| Race | 4 | 1.0543 |
| Education level | 2 | 1.0881 |
| Poverty-income ratio | 2 | 1.0608 |
| BMI | 2 | 1.0684 |
| Alcohol status | 2 | 1.0628 |
| Smoking status | 2 | 1.0891 |
| Diabetes | 1 | 1.0840 |
| Cardiovascular disease | 1 | 1.0531 |
| Hypertension | 1 | 1.1347 |
| Hyperlipidaemia | 1 | 1.0292 |
| Dietary zinc intake | 1 | 1.0450 |
| Serum copper | 1 | 1.1524 |
| ACE inhibitor use | 1 | 1.0728 |
| Diuretic use | 1 | 1.1060 |

Table S4. Events per variable for the fully adjusted logistic regression model

| CKD cases | Number of parameters | EPV |
| --- | --- | --- |
| 685 | 25 | 27.4 |

Table S5. hs-CRP-adjusted sensitivity analysis of serum zinc levels and odds of prevalent CKD restricted to NHANES 2015-2016

| Serum zinc levels (μmol/L) | Fully adjusted model + hs-CRP | |
| --- | --- | --- |
|  | OR (95% CI) | P value |
| Continuous | 0.990 (0.981-0.997) | 0.037 |
| Q1 [6.26, 10.91] | 1 [Reference] |  |
| Q2 (10.91, 12.29] | 0.97 (0.92-1.02) | 0.246 |
| Q3 (12.29, 13.82] | 0.97 (0.92-1.02) | 0.199 |
| Q4 (13.82, 35.57] | 0.94 (0.89-0.98) | 0.011 |
| P for trend |  | 0.014 |

The analysis was restricted to NHANES 2015-2016 participants with available hs-CRP data.

The fully adjusted model additionally included hs-CRP on top of the covariates in Model 3: age, sex, race/ethnicity, education level, poverty-income ratio, BMI, alcohol consumption, smoking status, diabetes mellitus, cardiovascular disease, hypertension, hyperlipidaemia, dietary zinc intake, serum copper, ACE inhibitor use, and diuretic use. Serum zinc was analyzed both as a continuous variable and as quartiles.

Table S6. Performance metrics of the 13 compared machine-learning models in the internal training and temporal test sets.

| Model | Dataset | AUC | Accuracy | Sensitivity | Specificity | Precision | F1 | Kappa | Weighted Brier score |
| --- | --- | --- | --- | --- | --- | --- | --- | --- | --- |
| XGBoost | Train/internal | 0.7887 | 0.8595 | 0.2848 | 0.9695 | 0.6414 | 0.3944 | 0.3280 | 0.0913 |
| Neural network | Train/internal | 0.7870 | 0.8610 | 0.2623 | 0.9755 | 0.6724 | 0.3774 | 0.3157 | 0.0908 |
| Logistic regression | Train/internal | 0.7857 | 0.8606 | 0.2287 | 0.9815 | 0.7034 | 0.3452 | 0.2891 | 0.0922 |
| Naive Bayes | Train/internal | 0.7807 | 0.8429 | 0.0291 | 0.9987 | 0.8125 | 0.0562 | 0.0456 | 0.1149 |
| LDA | Train/internal | 0.7790 | 0.8516 | 0.3027 | 0.9567 | 0.5720 | 0.3959 | 0.3203 | 0.0960 |
| Random forest | Train/internal | 0.7753 | 0.8573 | 0.1816 | 0.9867 | 0.7232 | 0.2903 | 0.2414 | 0.0933 |
| Ranger | Train/internal | 0.7730 | 0.8570 | 0.2152 | 0.9798 | 0.6713 | 0.3260 | 0.2689 | 0.0925 |
| QDA | Train/internal | 0.7674 | 0.7878 | 0.5247 | 0.8382 | 0.3830 | 0.4428 | 0.3156 | 0.1577 |
| C5.0 | Train/internal | 0.7653 | 0.8530 | 0.3027 | 0.9584 | 0.5819 | 0.3982 | 0.3239 | 0.0983 |
| KNN | Train/internal | 0.7483 | 0.8494 | 0.1480 | 0.9837 | 0.6346 | 0.2400 | 0.1908 | 0.1007 |
| Bagging | Train/internal | 0.7394 | 0.8483 | 0.2848 | 0.9562 | 0.5549 | 0.3763 | 0.3000 | 0.1038 |
| CART | Train/internal | 0.7180 | 0.8447 | 0.3004 | 0.9489 | 0.5296 | 0.3834 | 0.3023 | 0.1003 |
| SVM radial | Train/internal | 0.6752 | 0.8501 | 0.1570 | 0.9828 | 0.6364 | 0.2518 | 0.2010 | 0.1014 |
| Random forest | Test | 0.7987 | 0.8460 | 0.1715 | 0.9830 | 0.6721 | 0.2733 | 0.2198 | 0.0966 |
| XGBoost | Test | 0.7935 | 0.8573 | 0.3431 | 0.9618 | 0.6457 | 0.4481 | 0.3749 | 0.0955 |
| Logistic regression | Test | 0.7923 | 0.8559 | 0.2720 | 0.9745 | 0.6842 | 0.3892 | 0.3243 | 0.0948 |
| Neural network | Test | 0.7906 | 0.8608 | 0.3179 | 0.9711 | 0.6909 | 0.4355 | 0.3683 | 0.0933 |
| Naive Bayes | Test | 0.7903 | 0.8347 | 0.0209 | 1 | 1 | 0.0409 | 0.0343 | 0.1203 |
| LDA | Test | 0.7854 | 0.8488 | 0.3514 | 0.9498 | 0.5874 | 0.4397 | 0.3587 | 0.0985 |
| Ranger | Test | 0.7850 | 0.8524 | 0.2510 | 0.9745 | 0.6666 | 0.3647 | 0.3001 | 0.0957 |
| QDA | Test | 0.7768 | 0.7683 | 0.5857 | 0.8054 | 0.3794 | 0.4605 | 0.3215 | 0.1602 |
| KNN | Test | 0.7631 | 0.8403 | 0.1673 | 0.9771 | 0.5970 | 0.2614 | 0.2025 | 0.1032 |
| Bagging | Test | 0.7580 | 0.8390 | 0.2845 | 0.9516 | 0.5440 | 0.3736 | 0.2915 | 0.1072 |
| CART | Test | 0.7422 | 0.8249 | 0.2594 | 0.9397 | 0.4662 | 0.3333 | 0.2418 | 0.1152 |
| C5.0 | Test | 0.7278 | 0.8298 | 0.3556 | 0.9261 | 0.4942 | 0.4136 | 0.3172 | 0.1186 |
| SVM radial | Test | 0.7087 | 0.8403 | 0.1589 | 0.9788 | 0.6032 | 0.2517 | 0.1950 | 0.1068 |

Table S7. XGBoost tuning procedure and final hyperparameters

Table S7A. Candidate hyperparameter grid for XGBoost

| Parameter | Candidate values |
| --- | --- |
| Learning rate | 0.01, 0.05, 0.10 |
| Maximum tree depth | 2, 3, 4 |
| Minimum child weight | 1, 3 |
| Subsample ratio | 0.7, 1.0 |
| Column subsample ratio per tree | 0.7, 1.0 |
| Class-imbalance weight | Calculated from the ratio of negative to positive cases in the training set |
| Number of boosting rounds | Determined according to cross-validation performance |

Hyperparameter tuning was conducted within the training set only, using grid search. Candidate combinations were defined a priori. To address class imbalance, scale_pos_weight was set according to the ratio of negative to positive samples in the training set. The final number of boosting rounds was determined according to cross-validation performance. This table was provided to improve transparency and reproducibility of the exploratory machine-learning analysis.

Table S7B. Cross-validation performance of candidate XGBoost parameter combinations

| Rank | eta | max_depth | min_child_weight | subsample | colsample_bytree | best_iteration | mean CV AUC |
| --- | --- | --- | --- | --- | --- | --- | --- |
| 1 | 0.01 | 3 | 1 | 0.7 | 0.7 | 120 | 0.7894 |
| 2 | 0.05 | 3 | 3 | 0.7 | 1.0 | 39 | 0.7875 |
| 3 | 0.10 | 2 | 1 | 0.7 | 0.7 | 37 | 0.7833 |
| 4 | 0.05 | 3 | 1 | 0.7 | 0.7 | 66 | 0.7832 |
| 5 | 0.05 | 3 | 1 | 0.7 | 1.0 | 35 | 0.7829 |
| 6 | 0.01 | 4 | 3 | 0.7 | 0.7 | 29 | 0.7820 |
| 7 | 0.01 | 3 | 3 | 1.0 | 0.7 | 20 | 0.7814 |
| 8 | 0.10 | 3 | 1 | 1.0 | 0.7 | 29 | 0.7805 |
| 9 | 0.05 | 2 | 3 | 0.7 | 0.7 | 41 | 0.7804 |
| 10 | 0.10 | 2 | 3 | 0.7 | 0.7 | 26 | 0.7800 |

Candidate parameter combinations were ranked according to the mean cross-validated area under the receiver operating characteristic curve (AUC) in the training set. Only the top-ranked parameter combinations are shown here for clarity.

Table S7C. Final XGBoost hyperparameters used for model fitting

| Parameter | Final value |
| --- | --- |
| eta | 0.0100 |
| max_depth | 3.0000 |
| min_child_weight | 1.0000 |
| subsample | 0.7000 |
| colsample_bytree | 0.7000 |
| scale_pos_weight | 5.2242 |
| nrounds | 120.0000 |

The final XGBoost hyperparameters were derived from the grid-search and cross-validation procedure performed within the training set. These parameters were then used to fit the final XGBoost model for the exploratory machine-learning analysis and the SHAP-based feature attribution analysis.

Table S8. Focused validation summaries for the best-performing model and the XGBoost model used for SHAP analysis

Table S8A. Internal validation metrics of the best-performing model selected according to the test-set AUC

| Best-performing model | Dataset | AUC | Sensitivity | Specificity | Precision | F1 score | Kappa | Weighted Brier score |
| --- | --- | --- | --- | --- | --- | --- | --- | --- |
| Random forest | Train/internal | 0.7753 | 0.1816 | 0.9867 | 0.7232 | 0.2903 | 0.2414 | 0.0933 |
| Random forest | Test | 0.7987 | 0.1716 | 0.9830 | 0.6721 | 0.2733 | 0.2198 | 0.0966 |

The best-performing model was selected according to the test-set AUC among the candidate models evaluated under the same temporal split. Discrimination was summarized using AUC, sensitivity, specificity, precision, F1 score, and Cohen’s kappa. Calibration was summarized using the weighted Brier score. Calibration intercept and slope were explored but are not reported because weighted logistic recalibration was numerically unstable in the current dataset and returned non-informative outputs. This exploratory machine-learning analysis should be interpreted as supplementary to the survey-weighted regression analyses.

Table S8B. Discrimination and calibration metrics of XGBoost used for SHAP analysis

| Model | Dataset | AUC | Weighted Brier score |
| --- | --- | --- | --- |
| XGBoost | Test | 0.7935 | 0.0955 |

XGBoost was additionally evaluated because it was used for the SHAP-based feature attribution analysis shown in Figure 7. Stable quantitative metrics, including the test-set AUC and weighted Brier score, are reported here. Calibration intercept and slope were explored but are not reported because the weighted recalibration procedure remained numerically unstable and calibration-specific outputs were not fully consistent with the main performance file. Therefore, only stable discrimination and weighted Brier metrics are presented.
